# Galectin-3 deficiency in pregnancy increases the risk of fetal growth restriction (FGR) via placental insufficiency

**DOI:** 10.1038/s41419-020-02791-5

**Published:** 2020-07-23

**Authors:** Nancy Freitag, Irene Tirado-Gonzalez, Gabriela Barrientos, Katie L. Powell, Philipp Boehm-Sturm, Stefan P. Koch, Kurt Hecher, Anne C. Staff, Petra C. Arck, Anke Diemert, Sandra M. Blois

**Affiliations:** 1 Experimental and Clinical Research Center, a Cooperation between the Max Delbrück Center for Molecular Medicine in the Helmholtz Association, and Charité - Universitätsmedizin Berlin, Berlin, Germany; 2 Charité – Universitätsmedizin Berlin, corporate member of Freie Universität Berlin, Humboldt-Universität zu Berlin, and Berlin Institute of Health, Department for Psychosomatic Medicine, Berlin, Germany; 3 Laboratorio de Medicina Experimental, Hospital Alemán, Consejo Nacional de Investigaciones Científicas y Técnicas (CONICET), Buenos Aires, Argentina; 4 Division of Perinatal Research, Kolling Institute, Northern Sydney Local Health District, St Leonards, NSW 2065 Australia; 5 Charité – Universitätsmedizin Berlin, corporate member of Freie Universität Berlin, Humboldt-Universitätzu Berlin, and Berlin Institute of Health, Department of Experimental Neurology, Center for Stroke Research, Berlin, Germany; 6 NeuroCure Cluster of Excellence and Charité Core Facility 7 T Experimental MRIs, Charité – Universitätsmedizin Berlin, Berlin, Germany; 7 Department of Obstetrics and Fetal Medicine, University Medical Center Hamburg-Eppendorf, Martinistrasse 52, Hamburg, 20251 Germany; 8 Institute for Clinical Medicine, Faculty of Medicine, University of Oslo, Oslo, Norway; 9 Division of Obstetrics and Gyneacology, Oslo University Hospital, Oslo, Norway

**Keywords:** Reproductive disorders, Endocrine reproductive disorders

## Abstract

Fetal growth restriction (FGR) is the most common pregnancy complication in developed countries. Pregnancies affected by FGR, frequently concur with complications and high risk of neonatal morbidity and mortality. To date, no approved treatment is available for pregnant women affected with FGR. The objective of this study was to investigate the contribution of galectin-3 (gal-3), a β-galactoside binding protein involved in pregnancy, placental function and fetal growth. We demonstrated that lack of gal-3 during mouse pregnancy leads to placental dysfunction and drives FGR in the absence of a maternal preeclampsia syndrome. Analysis of gal-3 deficient dams revealed placental inflammation and malperfusion, as well as uterine natural killer cell infiltration with aberrant activation. Our results also show that FGR is associated with a failure to increase maternal circulating gal-3 levels during the second and third trimester in human pregnancies. Placentas from human pregnancies affected by FGR displayed lower gal-3 expression, which correlated with placental dysfunction. These data highlight the importance of gal-3 in the promotion of proper placental function, as its absence leads to placental disease and subsequent FGR.

## Introduction

During the establishment of a mammalian pregnancy, the placenta plays a critical role in controlling fetal-maternal resource allocation and mediating fetal programming of future disease. Placental function depends on a healthy decidual environment, and inadequate endometrial receptivity or inappropriate implantation increase the incidence of anomalous placentation. Defects in placentation often result in fetal growth restriction (FGR), one of the most complex pregnancy complications without currently available treatment aside delivery, most often preterm^1^. Affecting up to 8% of pregnancies worldwide, FGR is also associated with significant perinatal complications, such as increased risk of stillbirth, neonatal long-term morbidity, obesity, type 2 diabetes, and coronary disease later in life^1–4^. When not attributable to structural or genetic defects of the fetus, FGR is primarily caused by placental insufficiency^5,6^, but the biological processes that promote placental insufficiency and the subsequent progression to FGR are currently poorly understood. To date, no treatment options are available^1^.

Galectins, a family of soluble glycan-binding proteins are increasingly recognized as powerful modulators of pregnancy-associated processes including proper placental development. Among the galectin family members, the chimera-type galectin-3 (gal-3, encoded by the *Lgals3* gene) is highly expressed at the fetal-maternal interface^7–10^. Gal-3 knock-down in mouse endometrium results in substantially less implanted embryos^11^, and dysregulation of gal-3 is associated with several obstetrical complications resulting from placental dysfunction. These include preeclampsia (PE), hemolysis elevated liver enzymes and low platelets (HELLP) syndrome, small-for-gestational age, and gestational trophoblastic disease^12–16^. At present, the impact of dysregulated gal-3 expression in placental development and pregnancy outcome has not been addressed.

In this study, we found that gal-3 loss of function during gestation altered the decidual compartment favoring a pro-inflammatory milieu, which was accompanied by a decrease in progesterone (P4) in the maternal circulation. In the placental compartment, lack of gal-3 compromised placental vascularization and perfusion resulting in placental insufficiency and the subsequent development of asymmetric FGR in mice. Using the prospective birth cohort PRINCE (Prenatal Determinants of Children’s Health), we showed that development of FGR is accompanied by an altered kinetics of circulating maternal gal-3 levels during gestation. We also found that placental gal-3 expression is downregulated in human pregnancies complicated with FGR, demonstrating that gal-3 also plays a role in human FGR pathology. Finally, using reciprocal matings, we showed that gal-3 within the maternal compartment is required for proper placental development and fetal growth. Our findings identify gal-3 as a key component of the molecular program of decidual/placental development and offspring health, as well as a potential target for future strategies aimed at minimizing adverse outcomes in pregnancies at high risk for FGR.

## Results

### Gal-3 deficiency in pregnant mice leads to FGR

**Fig. 1:**
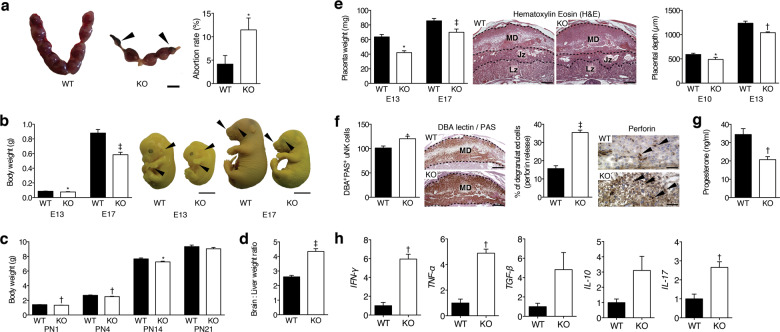
Gal-3 deficiency leads to FGR. **a** Macroscopic appearance of the implantation sites on embryonic day (E)13. The pictures show the normal phenotype of *Lgals3*^*+/+*^ (WT) compared to *Lgals3*^−/−^ (KO) dams, which exhibit resorbed fetuses (left panel, bar = 1 cm). Abortion rate (%) was calculated on E13 as follows: abortion rate = (fetal resorptions x 100)/total number of implantations (right panel, *n* = 19–24). **b** Fetal body weight (in grams) of E13 and E17 fetuses carried by *Lgals3*^*+/+*^ (WT) or *Lgals3*^−/−^ (KO) dams (left panel, *n* = 13–21). Theiler stage developmental analysis on E13, upper arrows show that pinna is not turned forward and lower arrows show absence of separated fingers in KO fetuses (bar = 0.25 cm). On E17, upper arrows display fewer skin wrinkles, smaller whiskers, and lower arrows show that eyes are clearly visible through the eyelids in gal-3 KO fetuses (bar = 0.5 cm) (right panel). **c** Mean body weights of pups from *Lgals3*^*+/+*^ and *Lgals3*^−/−^ dams. Reduced neonatal body weight was observed in *Lgals3* KO offspring, which persisted until postnatal day (P)14 (*n* = 55–161). **d** E17 asymmetrical FGR as identified by fetal brain-to-liver weight ratio (*n* = 15–30). **e** Placental weight (mg) observed on E13 and E17 (left panel, *n* = 13–21). H&E histological analysis of E10 and E13 implantation sites shows an enlarged decidua in *Lgals3*^−/−^ mice (middle panel, bar = 500 μm). Isolectin B4 (IB4) analysis of placental depth related to the decidua was significantly lower in placentas of *Lgals3* KO mice (right panel, *n* = 7–8). **f** Accumulation of mature NK cells (PAS^+^DBA^+^) were significantly elevated at E13 (left panel, bar = 500 μm, *n* = 15) and their perforin granules (arrows) were released in gal-3 deficient pregnancy (right panel, bar = 50 μm, *n* = 8–11). **g** Progesterone levels measured in E13 serum by ELISA (*n* = 5). **h** Quantitative PCR (qPCR) analysis of decidual *IFN-Ƴ*, *TNF-α*, *TGF-β*, *IL-10*, and *IL-17* gene expression in *Lgals3*^+/+^ (WT) and *Lgals3*^−/−^ (KO) dams at E7 (*n* = 4–9). In all figures, data are expressed as mean ± SEM. *P < 0.05, ^†^*P* < 0.01, and ^‡^*P* < 0.001 using two-tailed *t* test.

To evaluate the contribution of gal-3 during gestation, we analysed pregnancy outcome and fetal growth in gal-3 deficient mice. Although gestational length was similar between gal-3 wild type (*Lgals3*^*+/+*^) and deficient (*Lgals3*^−/−^*)* dams, gal-3 deficient dams displayed an increased frequency of fetal demise when compared to their wild type counterparts (Fig. 1a), which is in agreement with observations from other group^11^. Fetuses carried by *Lgals3*^−/−^ dams showed a weight reduction of 20–40%, compared with *Lgals3*^*+/+*^ fetuses during pregnancy, assesed on embryonic day (E)13 and E17 (Fig. 1b). The reduced fetal weight was accompanied by a delay in fetal development, as the majority of *Lgals3*^−/−^ fetuses only reached the Theiler stage (TS)21 on E13, whereas *Lgals3*^*+/+*^ offspring reached the TS22, which is appropriate for the gestational age. The immaturity of gal-3 deficient fetuses was still present on E17, evident as fewer skin wrinkles, smaller whiskers, and eyes visible through the eyelids corresponding to TS25 (Fig. 1b). In addition, we confirmed that *Lgals3*^−/−^ fetuses suffered from asymmetric FGR, as denoted by an increased brain-to-liver weight ratio (Fig. 1d). The reduction of fetal weight carried over into the postnatal period, as *Lgals3*^−/−^ offspring also showed a significantly reduced body weight until P14 (Fig. 1c). Upon weaning at 3 weeks of age, both *Lgals3*^−/−^ and *Lgals3*^*+/+*^ offspring displayed similar body weights (Fig. 1c).

### Lack of gal-3 during murine gestation is linked to features of placental insufficiency

Since asymmetric fetal growth primarily arises from placental pathologies^17^, we next investigated whether the lack of gal-3 causes placental insufficiency. We observed reduced placental weight on E13 and E17 in *Lgals3*^−/−^ mice (Fig. 1e). Furthermore, in contrast to wild type dams, the placental disc in E10 and E13 *Lgals3*^−/−^ placentas displayed reduced trophoblast layers with a correspondingly enlarged maternal decidua (Fig. 1e). Improper placental development in *Lgals3*^−/−^ mice was accompanied by a significantly increased abundance of uterine NK (uNK) cells in the decidua. Here a higher frequency of uNK cells contained cell-free, perforin-reactive granules (Fig. 1f). As shown in Fig. 1g, gal-3 deficiency was associated with a significant reduction in P4 levels. In line with this finding, mRNA expression of inflammatory cytokines (e.g. INF-γ, TNF-α, and IL-17) was significantly increased in *Lgals3*^−/−^ decidual tissue (Fig. 1h).

**Fig. 2:**
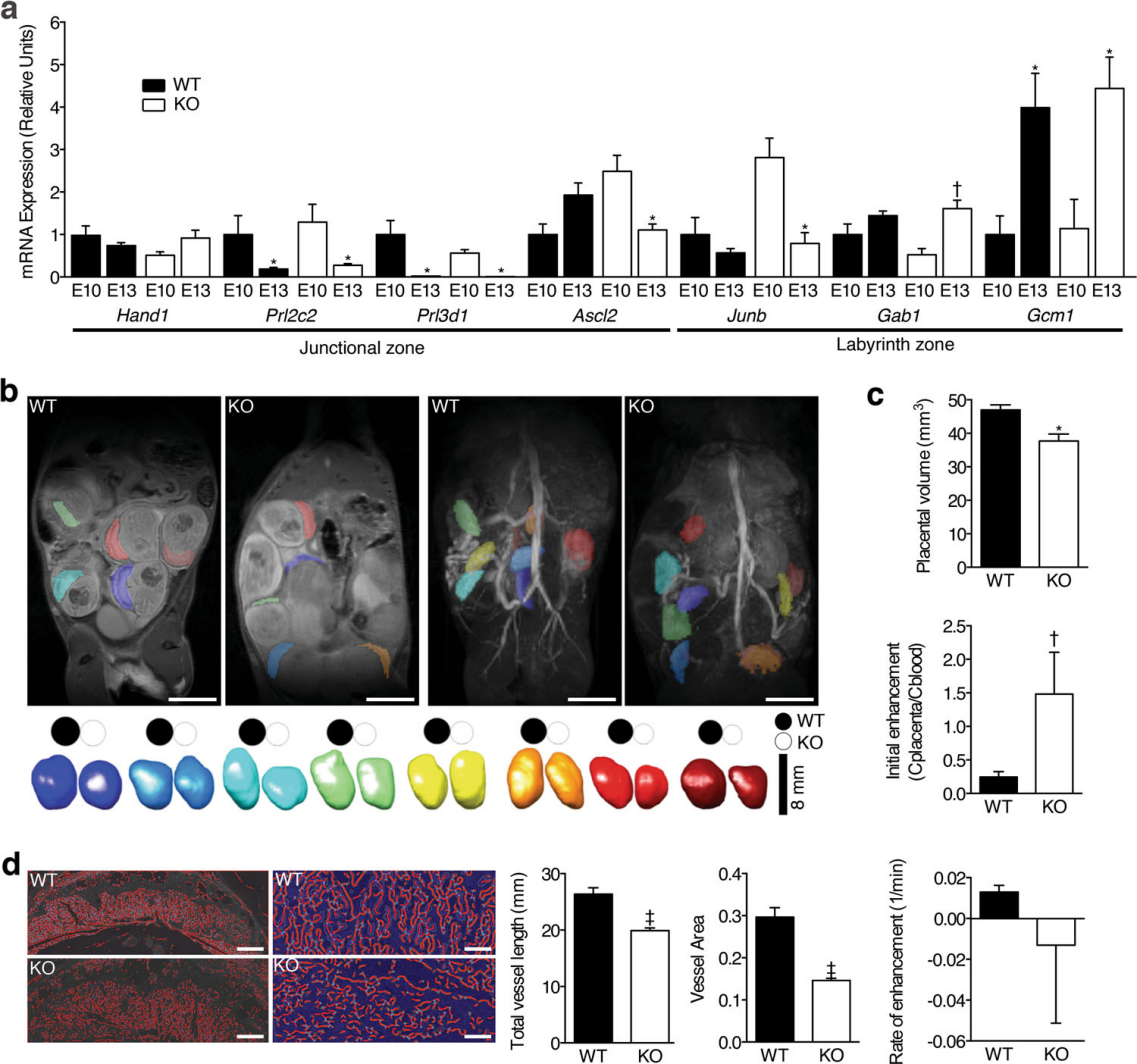
Gal-3 deficiency results in placenta insufficiency. **a** Relative mRNA expression of trophoblast differentiation genes determined by quantitative PCR (qPCR) on E10 and E13 placentas (*n* = 6–8). **b** 2D pre-contrast anatomical overview of MR images through abdomen of representative *Lgals3*^*+/+*^ (WT) and *Lgals3*^−/−^ (KO) dams. Placentae labeled with false color transparent overlay (top left panel). 3D post-contrast maximum intensity projection angiograms of the mouse abdomen to determine placental volumes based on contrast enhancement (top right panel). 3D reconstruction of individual placentas ordered by volume (largest to smallest from left to right) are shown pair wise for *Lgals3*^*+/+*^ (WT) and *Lgals3*^−/−^ (KO) animals. For better visual comparison, placental volumes were projected onto discs shown above each reconstruction illustrating smaller placentas in the *Lgals3*^−/−^ animals (bottom panel, bar = 8 mm). **c** Group comparison of placental volumes over all animals confirmed a decrease in *Lgals3*^−/−^ animals (top panel). Series of post-contrast images were acquired and mean signal was quantified repetitively for each placenta over ~10 min for functional MRI phenotyping. Initial enhancement, representative of maternal fractional blood volume in percent was increased in *Lgals3*^−/−^ placentae whereas the rate of enhancement representative of several functional parameters such as perfusion, contrast agent inflow, clearance, and uptake was unchanged. Notably, a higher variability was found in KO animals for both, initial (middle panel) and rate (bottom panel) of enhancement (*n* = 8–16). **d** Isolectin B4 (IB4) staining showed poor development of fetal capillaries in the labyrinth of placentae of *Lgals3*^−/−^ KO mice as revealed by AngioTool analysis (Total vessel length and vessel area; left bar = 500 μm and right = 150 μm, *n* = 25–34). In all figures, data are expressed as mean ± SEM. *P < 0.05, ^†^*P* < 0.01, and ^‡^*P* < 0.001 using two-tailed *t* test.

Placental gal-3 deficiency may also affect trophoblast differentiation during placentation. To address this possibility, we examined the expression of trophoblast cell markers between E10 and E13. Our analysis revealed altered expression of the trophoblast *Junb*, *Gab1*, and *Gcm1* genes, a phenomenon that likely contributed to the failure of placental labyrinth differentiation in *Lgals3*^−/−^ placentas (Fig. 2a). To further examine the placental labyrinth structure, we performed morphological and functional magnetic resonance imaging (MRI) during E13. As illustrated in Fig. 2b, placental volume was reduced in *Lgals3*^−/−^ compared to *Lgals3*^*+/*+^ implantations. After injection of an albumin-bound contrast agent, initial contrast enhancement was significantly higher for *Lgals3*^−/−^ mice but contrast material dynamics described by rate of enhancement were similar (Fig. 2c). Interestingly, the variability of both parameters was much higher in *Lgals3*^−/−^ animals. Finally, we used Isolectin B4 staining to further characterize the *Lgals3*^−/−^ placental labyrinth zone. As expected, gal-3 deficiency resulted in reduced placental labyrinth total vessel length and vessel area (Fig. 2d).

### Reduced maternal levels of gal-3 are linked to human FGR

**Fig. 3:**
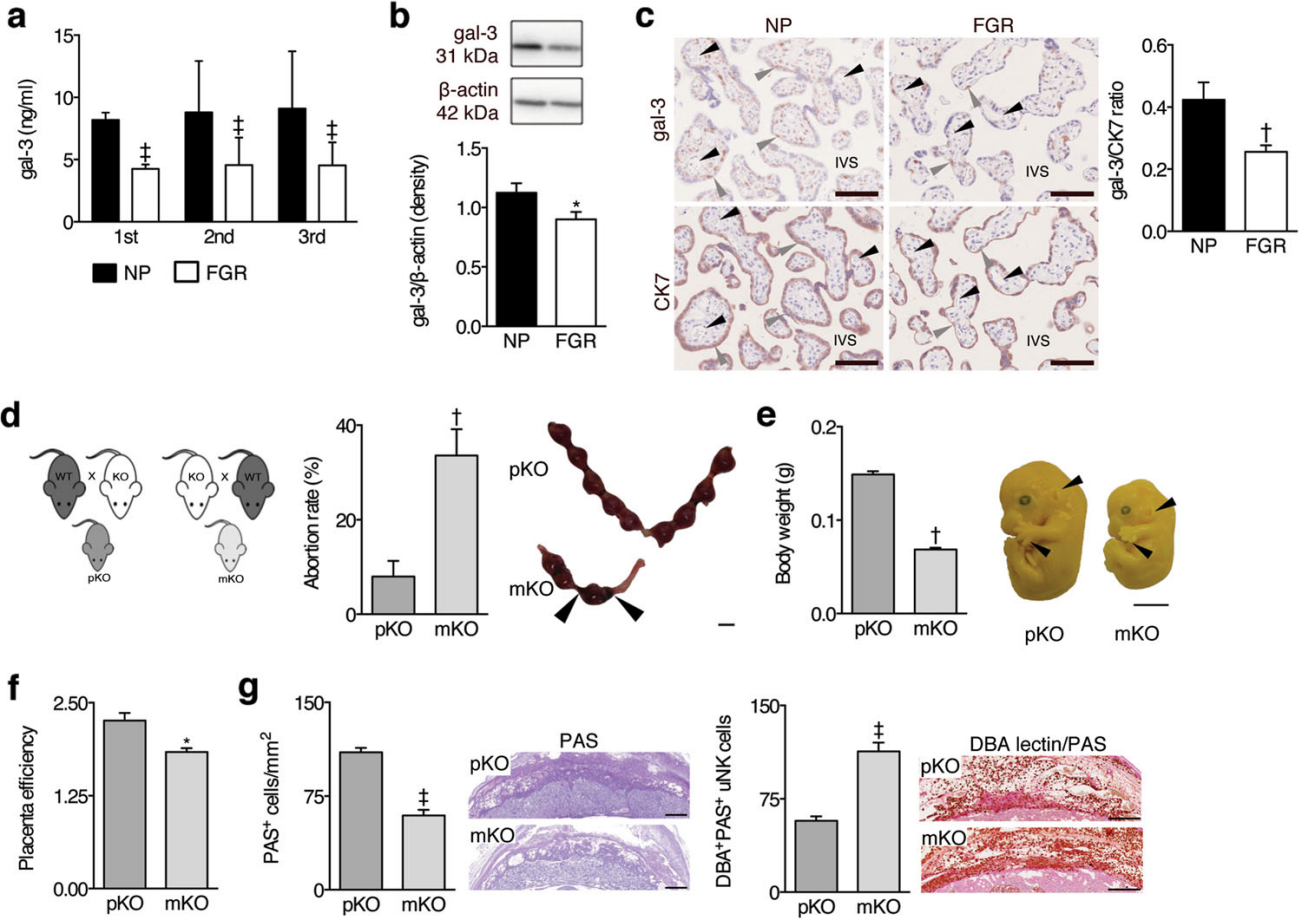
Maternal gal-3 is necessary for normal placental and fetal development. **a** Circulating maternal gal-3 evaluated by ELISA in PRINCE cohort of patients (*n* = 65) during first, second, and third trimester, where some women subsequently development FGR (*n* = 32). **b** Western blot analysis of gal-3 expression in placental tissue from normal, healthy human pregnancies (NP) (*n* = 27) and pregnancies complicated by FGR (*n* = 19). Densitometry was performed to quantify changes in protein expression using ImageJ. **c** Representative images of gal-3 and cytokeratin (CK) 7 immunohistochemistry in NP and FGR placentae (*n* = 14, bar = 100 μm; left panel). The percent staining area of gal-3 and CK7 slides was quantified, and a ratio was calculated by dividing the percent area of gal-3 to CK7 staining in slides from the same placental tissue sample (right panel). **d** Schematic diagram showing the lack of paternal (mating of *Lgals3*^−/−^ (KO) male with *Lgals3*^*+/+*^ (WT) female, pKO) and maternal (mating of *Lgals3*^*+/+*^ (WT) male with *Lgals3*^−/−^ (KO) female, mKO) gal-3. The offspring is heterogeneous (HT) for gal-3 in both mating combinations (left panel). The abortion rate was calculated on E13 as follows: abortion rate = (fetal resorptions x 100)/total number of implantations (middle panel, *n* = 6–8). Pictures display whole implantation sites on E13. Arrows point to fetal resorptions (right panel, bar = 1 cm). **e** The embryo body weight was determined on E13 to identify potential fetal growth restriction (FGR) (*n* = 6). To assess the fetal development, Theiler stage (TS) analysis was performed. Normally developed embryos on E13 are in TS22 (pKO). FGR embryos are in a lower stage (e.g., TS21 or 21.5) and are characterized by a pinna that is not turned forward and non-separated fingers (arrows; mKO) (bar = 0.25 cm). **f** Placental efficiency was calculated as fetal/placental weight ratio on E13 (*n* = 14–21). **g** Glycogen cells in the spongiotrophoblast of the placenta were stained with periodic acid Schiff (PAS) and counted in 3 squares (1 × 1 mm) per mouse on E13 (*n* = 14–21). The number of mature uterine natural killer (uNK) cells in the decidua basalis were counted in 3 to 4 squares (1 × 1 mm) per mouse (n = 19–26). In all figures, data are expressed as mean ± SEM. *P < 0.05, ^†^*P* < 0.01, and ^‡^*P* < 0.001 using two-tailed *t* test.

We next sought to asses the translational relevance of our findings in mice and determined gal-3 levels in human pregnancies using biological samples from two independent patient cohorts. In a subgroup of women participating in the prospective pregnancy cohort PRINCE carried out in Germany (clinical data shown in Supplementary information), we observed that serum gal-3 levels continuosly increased in normally progressing pregnancies (*n* = 33) (range 3.02–16.70 ng/ml, Fig. 3a). Conversely, in pregnant women affected by FGR (*n* = 32), levels of serum gal-3 were significantly lower as early as the first trimester and levels stalled throughout gestation (range 1.00–9.79 ng/ml). In order to asses the interaction between serum gal-3 levels in human pregnancies and placental function, we analysed its correlation with uterine artery perfusion (Ut PI). As suspected, gal-3 peripheral levels were found to correlate inversely with Ut PI values (*r*_*s*_ = −0.5065*, P* < 0.05) in pregnancies complicated with FGR. In addition, we used placental biopsies taken at birth from uneventful (*n* = 27) and FGR pregnancies with placental pathology (*n* = 19) in an independent cohort recruited in Australia (clinical data shown in Supplementary information,^18^). Similar to our observations in mice, FGR placentas expressed decreased protein levels of gal-3 in general as revealed by western blot (Fig. 3b), or when specifically assessed within the extravillous CK7^+^ trophoblasts (Fig. 3c, Supplementary Fig. 1).

Since placental insufficiency is not exclusive to FGR, we wondered whether gal-3 dysregulation also occurs in pregnancies complicated by preeclampsia. Placental expression and circulating levels of gal-3 in preeclamptic women (*n* = 36) from the Oslo study were not different from those of uneventful pregnancies (*n* = 23) (Supplementary Fig. 2a–d; clinical data shown in Supplementary information). Supporting this, gal-3 deficient mice did not develop preeclampsia-like features (increased blood pressure, new-onset proteinuria, production of Angiotensin II receptor type 1 autoantibody (AT1AA) or circulating anti-angiogenic factors (Supplementary Fig. 2e–h). In addition, no differences were found in E10 and E13 decidual spiral artery wall thickness between WT and KO dams (Supplementary Fig. 2i). We conclude that gal-3 deficiency is involved in the placental-fetal axis and confined to the fetal phenotype downstream of placental pathology without significantly affecting maternal vascular health in pregnancy.

### Maternally-derived gal-3 drives proper placental function and fetal growth

We next investigated whether maternal gal-3 plays a role in placental and fetal development. As shown in Fig. 3d, absence of maternal gal-3 increased fetal demise. Most notably, we observed that the body weight of *Lgals3*^+/−^ fetuses derived from *Lgals3*^−/−^ dams (maternal KO, mKO) was significantly reduced compared to *Lgals3*^+/−^ fetuses carried by *Lgals3*^*+/+*^ dams (paternal KO, pKO). Also, an immature Theiler stage (TS21), characterized by a backwards pinna and unseparated fingers (Fig. 3e), was detected in fetuses derived from *Lgals3*^−/−^ dams. In addition, *Lgals3*^+/−^ implantations carried by *Lgals3*^−/−^ dams displayed a decreased fetal/placental weight ratio, indicative of placental insufficiency (Fig. 3f), along with a reduction of PAS^+^ glycogen cells in the junctional zone (Fig. 3g, Supplementary Fig. 3a). We also identified an increased uNK cell abundance in the decidua of *Lgals3*^−/−^ dams, compared to the reciprocal matings (Fig. 3g, Supplementary Fig. 3b). These findings highlight that maternally-derived gal-3 maintains placental function. If maternal gal-3 expression is low, the incidence of developing FGR is increased.

## Discussion

FGR remains one of the “great obstetrical syndromes”, and is a leading contributor worldwide to perinatal mortality and morbidity, as well as playing an important role in mediating non-communicable diseases in adult life, including cardiovascular disease, diabetes, metabolic syndrome, and obesity. Although significant advances have been made on its understanding, there is still no definitive cure or treatment for pregnancies complicated by FGR. Thus, the current knowledge gap emphasizes the need for a better understanding of how fetal development is normally achieved and how it is dysregulated in FGR. Since normal placental development and functional integrity are essential for normal fetal growth, the identification of alterations during critical windows of placental development could help to optimize antepartum monitoring and timely delivery of FGR infants. Our study unveils that a dysregulation of gal-3, especially at the maternal compartment, is a significant factor involved in abnormal placentation and development of FGR. This phenotype appears to be mirrored in human FGR patients, suggesting that gal-3 can assert similar effects in the human placenta.

Galectin-3 was initially reported to be a factor related to endometrial receptivity due to its increased expression in the endometrium and trophoblast during embryo implantation^19,20^. Subsequent studies have revealed that endometrial gal-3 is involved in embryo implantation^21^ and dysregulation of placental gal-3 is associated with adverse pregnancy outcomes^12–16,22,23^. The present study demonstrates that gal-3 is critical for orchestrating healthy fetal-maternal interface interactions and its dysregulation results in asymmetric FGR. Affecting approximately 70% of growth-restricted fetuses, the asymmetric type of FGR occurs when brain growth and maturity is maintained while the visceral organs (especially the liver) have relatively reduced weights^24^, and represents a later timing of embryonic damage that results from maternal vascular factors and/ or placental insufficiency. The fact that gal-3 deficiency-induced asymmetric growth restriction followed by relatively rapid catch-up growth is of significant importance since human babies with these characteristics, particularly those with accelerated postnatal weight gain after in utero growth restriction, have a greatly enhanced risk of developing type 2 diabetes, obesity and cardiovascular disease^25–27^.

Studies in gal-3 deficient dams enabled us to identify this chimera lectin as a key player in regulating decidualization and maternal immune adaptation during early gestation. The absence of gal-3 *per se* induced decidual enlargement, inflammatory cytokine gene expression, and NK cell infiltration with aberrant activation. Supporting our observations, these features were also found in a different mouse FGR model established by independent researchers^28^. Gal-3 is known to regulate immune responses, acting as a pro-inflammatory signal with diverse innate immune cell targets to promote their activation, degranulation, and cytokine production^29,30^. In this context, recent *in vitro* studies have shown that endogenous expression of gal-3 in human NK cells can be stimulated by activating cytokines (IL-2, IL-15) and correlates functionally with their degree of cytotoxic degranulation^31^. While similar functions remain to be examined in the context of pregnancy, the identification of an endogenous gal-3 ligand localizing specifically to mouse uNK perforin granules^32^ implies a potential role of this lectin in controlling the activation status of this particular lymphocyte subset, which has been ascribed important roles in the modulation of decidual development, trophoblast invasion, and maternal vascular remodeling during early pregnancy.

Gal-3 deficiency impacts placental function by means of intrinsic effects on trophoblast biology. Specifically, we showed that gal-3 deficiency during gestation alters trophoblast differentiation, vascularization of the labyrinth zone and subsequently causes changes in placental perfusion. While the structural organization of the fetal-maternal interface differs between human and mouse (villous versus labyrinthine placenta), the syncytium separates fetal and maternal blood in both species and serves analogous functions. Therefore, it is not surprising that gal-3 deficiency compromised the labyrinth layer during mouse placentation. Since inflow and clearance of the contrast agent strongly depend on perfusion, we speculate on more heterogeneous maternal circulation in gal-3 deficient compared to control placentas. Indeed, gal-3 deficiency caused reduced total vessel length and vessel area in the placental labyrinth, suggesting that absence of this lectin during gestation alters vascularization of the labyrinth and subsequently results in placental malperfusion. On the other hand, maternal deficiency of gal-3 also provoked alterations in placental structure, particularly in glycogen cells of the junctional zone, emphasizing the importance of an interplay between fetal and maternal sources of gal-3 expression for normal placental development. Alterations in placental energy storage (glycogen cells) are a typical feature of FGR models and likely to contribute to the detrimental maternal effect on fetal growth^33,34^. A reduced placental glycogen reserve will definitely impact on placental well-being and fetal growth as the main source of energy for the placenta comes from glucose and also, the glycogen stores may be destined to the fetal demands^35^. In addition, glycogen trophoblasts may serve other functions likely to impact fetal growth, as these cells invade the decidua congregating near spiral arteries to support their remodeling and also have an endocrine role, being the source of several mediators including retinoic acid, prolactin-like hormones and particularly IGF-2, a crucial modulator of placental and fetal growth^33,36^.

Finally, our results showing dysregulated gal-3 expression in human pregnancies affected by impaired fetal growth are consistent with the role played by this lectin in pregnancy orchestration as demonstrated in our mouse studies. Particularly, the fact that besides sharing a common placental histopathology, FGR and PE placentas differed in their pattern of regulation of gal-3 expression is of great importance. Our clinical and experimental findings demonstrated that gal-3 loss of function is not sufficient to provoke a maternal PE-like syndrome in mice, and women with both early and late-onset PE showed no alterations in gal-3 expression. Because gal-1 and gal-3 are the most prominent galectins associated with critical processes in early gestation, we hypothesize that unique properties exerted by gal-3 in supporting placental development, fetal growth, and pregnancy cannot be substituted by gal-1. Our study is clinically relevant as it provides important insights into the role of gal-3 during gestation, highlighting its requirement for proper placental development and function and fetal growth. It also reinforces the concept that unique functional properties in support of healthy pregnancy are specific to each of the different members of the placental galectin network.

## Materials and methods

### Mice

C57BL/6 *Lgals3*^*+/+*^ and *Lgals3*^−/−^ mice were purchased from Jackson Laboratories. The presence of a vaginal plug after mating was denoted as embryonic day (E) 0. Timed pregnant *Lgals3*^+/+^ and *Lgals3*^−/−^ mice were evaluated at E7, E10, E13, E17, P4, and P21 (*n* = 7–9 mice per group) and approved by Charité and Berlin authority for Animal Use in Research and Education. A second experiment used *Lgals3*^*+/+*^or *Lgals3*^−/−^ females mated with *Lgals3*^−/−^ or *Lgals3*^*+/+*^ males respectively to create the “paternal” (pKO) or “maternal” (mKO) gal–3 deficiency. On E13, after euthanizing the mice, the percentage of failed implantations was calculated (non-viable implantations/total implantation ×100) and whole implantation sites were frozen for histological sectioning and isolation of total protein or total RNA according to our published procedures^7^. Embryos were fixed in Bouin’s solution and subsequently cleared in 70% v/v ethanol for body weight measurement and Theiler stage analysis^37^. Placenta efficiency was calculated as “fetal/placental weight ratio” and used to measure nutrient transfer capacity from the placenta to the fetus.

### Human samples

Three patient cohorts were used in this study. The prospective human low-Risk pregnancy cohort PRINCE (Prenatal Identification of Children’s Health, Ethics Committee: PV3694) is based at the university medical center in Hamburg, Germany. Since 2012 a total number of 721 women have been included during early pregnancy and examined a total of three times during the course of the pregnancy (14th, 24th, and 36th week of pregnancy). In addition to collecting demographic details and psychosocial factors, these prenatal study visits involved extensive ultrasound examinations of fetal growth and placental function, nutritional behavior, medication intake, and vaccinations during pregnancy. Blood was drawn at each of the three-time points.

For analyses of gal–3 expression in pregnancy affected only by FGR, patient samples from the research bio-bank collection at the Kolling Institute, Sydney, Australia were used. Use was approved by the Northern Sydney Local Health District Human research Ethics Committee, (St Leonards, NSW, Australia) and was assigned the site-specific assessment number 0912-348 M and the Australian national ethics application form number HREC/09/HARBR/165 as described^18^.

For analyses of gal–3 expression in pregnancy affected by preeclampsia, patient samples from the Oslo Pregnancy Biobank research collection at Oslo University Hospital, Oslo, approved by the Regional Committee of Medical Research Ethics in Eastern Norway, were used as described^38^. Circulating soluble fms-like tyrosine kinase-1 (sFlt-1) and placenta growth factor (PlGF) levels were determined by commercial ELISA from R&D Systems following the manufacturer’s recommendations. The clinical characteristics of the human cohorts are summarized in Supplementary information.

### Histological analysis

Serial paraffin-embedded uterine sections from *Lgals3*^*+/+*^ and *Lgals3*^−/−^ mice at E13 were cut into 4 μm-thick sections and stained with hematoxylin-eosin (H&E), PAS, Masson-Goldner’s trichrome, Dolichos biflorus agglutinin (DBA) lectin/PAS and Isolectin B4 as previously described^39^. Whole implantation sites were stained and digitally scanned by a high-resolution bright field and fluorescence slide scanner (Pannoramic MIDI BF/FL, 3DHISTECH Ltd.), and staining was evaluated on virtual slides using Pannoramic Viewer 1.15.4 (3DHISTECH Ltd.) by two examiners blinded to the pregnancy outcome.

### Magnetic resonance imaging

MRI measurements for in vivo phenotype of *Lgals-3*-deficient mice at E13 were performed in a dedicated 7 T small animal scanner (Biospec, Bruker BioSpin, Ettlingen, Germany). The imaging protocol was adapted from a previous study by Plaks and coworkers^40^. Full details on the MRI protocol is provided in the Supplementary information.

### Galectin-3 ELISA

Gal-3 concentrations in the serum of pregnant patients were determined by ELISA as described previously^41^. Briefly, immunolon 2 ELISA plates (Dynatech Laboratories, USA) were covered with polyclonal antihuman gal-3 antibody (1 μg/ml; AF1154, R&D Systems, USA) and washed with washing buffer (0.5% Tween-20 in PBS). Plates were blocked with 3% BSA in PBS. Individual wells were incubated with serial dilutions of gal-3 (1154-GA, R&D Systems, USA) or serum samples (diluted 1/10) for 2 h at RT. Wells were washed and incubated with biotinylated polyclonal antihuman gal-3 antibody (0.25 μg/ml in PBS 0.1% BSA; BAF1154, R&D Systems, USA). Plates were washed six times and incubated with horseradish peroxidase (HRP)-conjugated streptavidin (Calbiochem, USA). After eight additional washes, a colorimetric reaction was developed with the 3,3,5,5′-tetramethyl benzidine (TMB) substrate (Pierce Biotechnology, USA). The reaction was stopped by adding one volume of 4 N H_2_SO_4_. Absorbance at 450 nm was recorded. Each reported value is the mean of triplicate assays.

### Gal-3 western blotting

Protein was extracted from frozen placental tissue and 10 µg of protein lysate was separated by SDS-PAGE as described previously^19^. Immunoblotting was performed for gal-3 (1:1000, Santa Cruz Biotechnology SC-20157) or β-actin (loading control) and protein expression was detected using the appropriate HRP-conjugated secondary antibodies (1:3000, Bio-Rad).

### Galectin-3 staining in human samples

Immunohistochemistry was performed on formalin-fixed, paraffin-embedded placental tissues from both normal and FGR pregnancies as previously described^19^. Slides were incubated with gal-3 (0.5 µg/ml; Santa Cruz Biotechnology sc-32790), cytokeratin 7 (0.09 µg/ml; Abcam ab68459) or isotype controls at equivalent concentrations, visualized using NovaRed peroxidase HRP substrate kit (Vector Laboratories) and staining quantitated using ImageJ.

### Statistical analysis

Data are expressed as mean ± SEM. Mouse and human data were analysed using the non-parametric Mann–Whitney *U*-test. GraphPad Prism 8.0 (GraphPad Software, Inc.) was used to analyse the data and a *P* value <0.05 was considered as statistically significant.

## Supplementary information

Supplementary information

Supplementary information 2

Supplementary information 3

Supplementary information 4
