# Supplementary material for: Galectin-3 deficiency in pregnancy increases the risk of fetal growth restriction (FGR) via placental insufficiency
Source: Cell Death Dis. 2020 Jul 23;11(7):560. doi: 10.1038/s41419-020-02791-5 (PMC7378206; doi:10.1038/s41419-020-02791-5)
Supplement: Supplementary file 1 — Supplementary information [file 41419_2020_2791_MOESM1_ESM.docx]

**Supplementary Information**

**Materials and Methods**

**Human Cohorts**

For analyses of serum gal-3 levels in human pregnancies we selected women from the low-risk pregnancy study PRINCE (n = 721) with a low estimated fetal weight, that was later confirmed at birth to be below the 10^th^ percentile (n = 37). Although most cases have shown some form of pathological doppler increased pulsatility index in the uterine or umbilical arteries, these were considered FGR for this analysis irrespective of doppler values. Pregnancies received after assisted reproductive technology, multiples or fetuses with a suspected chromosomal or structural abnormality were excluded from this cohort. Furthermore, women were excluded that smoked, reported any illicit drug use or underlying severe medical condition that required constant medication. From the appropriate grown fetuses of the PRINCE cohort we matched 37 cases by maternal age, maternal BMI in the first trimester and fetal sex. Participants were seen at least once per trimester around gestational age 14, 24 and 34 weeks of gestation and received among other a detailed ultrasound examination that included estimated fetal weight (according to Warsof in the first trimester and according to Hadlock 2 in the second and third trimester). According to ISUOG standards they received a doppler examination of the umbilical and uterine arteries using a GE Voluson E8 Expert machine. Women provided written informed consent and the study was evaluated by the Ethical committee of the chamber of physicians in Hamburg, Germany (PV 3694). For clinical characteristics of the participants please refer to **Table 1.**

For analyses of placental gal-3 expression in pregnancy affected only by FGR, patient samples from the research bio-bank collection at the Kolling Institute, Sydney, Australia were used (**Table 2**). FGR was diagnosed prior to delivery via ultrasound where the estimated fetal weight was ˂10^th^ percentile for gestational age and was associated with abnormal Doppler ultrasound reading, weight was confirmed at birth. No small for gestational age (SGA) placentas were included in our FGR cohort. Women provided written informed consent prior to delivery for collection of human placental tissue. Placentas were collected from non-labouring or minimally labouring women following delivery by lower caesarean section from pregnancies complicated by FGR or gestational age and BMI matched control pregnancies from 27-41 weeks gestation. Women with healthy normal pregnancies either elected to have C-sections close to and at term, others delivered pre-term (prior to 38 weeks gestation) were due to vasa previa or medical advice due to previous C-section. Exclusion criteria included smoking, pre-existing or gestational hypertension and preeclampsia. FGR was diagnosed prior to delivery based on previously reported criteria ^1^. Following collection, placental tissue was washed in sterile saline and either snap frozen in liquid nitrogen for extraction of RNA/protein or fixed in 10% (v/v) neutral buffered formalin for subsequent paraffin embedding.

For analyses of gal-3 expression in pregnancy affected by preeclampsia, patient samples from the Oslo Pregnancy Biobank research collection at Oslo University Hospital, Oslo (**Table 3**), approved by the Regional Committee of Medical Research Ethics in Eastern Norway, were used as described ^2^. Informed written consent was obtained from each participant. Placental biopsies from central non-infarcted cotyledons were obtained following caesarean sections from 35 preeclamptic women and 36 women with normotensive and uncomplicated pregnancies. Decidual tissue was collected through vacuum suctioning of the placental bed after gentle placental delivery (6). Only women with singleton pregnancies were included, and none had pre-existing hypertension, chronic diseases, rupture of membranes, clinical signs of infection, or were in labor at blood sampling. Preeclampsia was defined as blood pressure augmentation after 20 weeks gestation to >140/90 mmHg on two or more occasions 6hour apart in a previously normotensive woman, combined with proteinuria. Proteinuria was defined as protein dip stick ≥1+ on two or more midstream urine samples 6 hour apart or a 24hour urine excretion of ≥0.3 g protein in the absence of urinary infection (7). The uncomplicated pregnancy group consisted of healthy, normotensive women undergoing caesarean section due to breech presentation or other reasons.

**Magnetic Resonance Imaging**

For in vivo phenotyping of *Lgals3^-/-^* mice at E13 during pregnancy, animals underwent MRI measurements in a dedicated 7T small animal scanner (Biospec, Bruker BioSpin, Ettlingen, Germany) using a 40 mm diameter volume resonator coil for transmission and reception (Bruker). Anesthesia was induced using 3% isoflurane in a mixture of 70% N_2_O and 30% O_2_ and animals were fixed in supine position on an animal holder using a tooth bar and adhesive tape. Isoflurane was reduced to 1.5%-2% to maintain anesthesia. Body temperature was measured using a rectal probe and maintained at 37.0°C±0.5°C using a warm water blanket and a pressure-sensitive pad was placed on top of the thorax to monitor respiration rate using MR compatible equipment (SA Instruments, Stony Brook, NY, USA). The imaging protocol was adapted from a previous study by Plaks and coworkers ^3^. *Lgals-3^-/-^* (n = 5, n = 42 placentas) and wild type control animals (n = 5, n = 36 placentas) underwent anatomical imaging using a respiration-triggered T2 weighted 2D RARE sequence (20 contiguous slices, 1 mm slice thickness, 0.2 mm slice gap, field of view FOV = 39.2 x 51.2 mm^2^, matrix MTX = 196 x 256, echo spacing ΔTE = 8 ms, effective echo time TE = 16 ms, repetition time TR = 1.6 s, RARE factor 8, flip angle FA = 90°/180°, 6 averages, time of acquisition TA = 3:50 min, effective TA due to triggering ~ 5-7 min). Dynamic contrast enhanced (DCE) MRI was carried out after tail vein injection of 12 mg/mouse of BSA-Gd-DTPA (SyMO-Chem B.V., Eindhoven, The Netherlands) in a 200 µl PBS chase. Pre-contrast T1 mapping was carried out using five variable flip angle 3D FLASH images (FOV = (51.2 mm)^3^, MTX = 128 x 128 x 64 zerofilled to 128^3^, TE = 3 ms, TR = 10 ms, FA = 5°/15°/30°/50°/70°, 2 averages, TA = 2:44 min per FA) fitted by a linearized model based on ROCKETSHIP scripts ^4^(<https://github.com/petmri/ROCKETSHIP)>. This was followed by ten post-contrast DCE images acquired with the same pulse sequence but fixed FA = 30° (TA = 27:18 min). Only the first four post-contrast DCE images were used for analysis due to a violation of the underlying DCE model since we observed a previously described secondary enhancement, which is likely linked to active trophoblast uptake of contrast material ~15 min post injection ^3^. On DCE images, a volume of interest (VOI) for each placenta was semi-automatically segmented on the third image post injection using the ITK Snap (www.itksnap.org) 3D segmentation tool based on contrast enhancement ^5^. A blood VOI was drawn in a caudal part of the aorta. VOIs were manually adapted for all other images to correct for motion between scans. Placenta volume in mm^3^ was extracted from the first image post injection. DCE parameter fitting of initial enhancement (contrast agent concentration in the placenta divided by blood C_placenta_/C_blood_) and rate of enhancement (ROE in min^-1^) were carried out in custom MATLAB 2014a (MathWorks, Natick, MA USA) scripts using the previously described models ^3^.

**VEGF and soluble endoglin (sEng) ELISA**

The VEGF DuoSet® ELISA (R&D Systems, DY493) and mouse Endoglin DuoSet® ELISA (R&D Systems, DY3120) were used to measure VEGF and sEndoglin serum levels following the manufacturer’s recommendations. A microtiter plate (Nunc-Immuno™ Plate, 456537) was coated with capture the capture antibody overnight. All incubations were performed at room temperature. After washing the plate, blocking was performed by adding 1% BSA in PBS. The plate was washed and the standard, prepared in a 2-fold dilution series (from 1000 to 15.625 pg/ml VEGF; from 4000 to 62.5 pg/ml endoglin), and mouse serum samples in a 2-fold dilution were applied and incubated 2hours. After washing, the detection antibody was incubated 2 hours followed by Streptavidin-HRP for 20 minutes. After washing, a 3,3’,5,5’-Tetramethylbenzidine (TMB) substrate solution was added and incubated for 20 minutes in the dark. The colorimetric reaction was stopped with 4N H_2_SO_4_ and the optical density (OD) was determined at 450 nm. The VEGF and sEng serum levels were calculated from the standard curves with a four-parameter logistic (4-PL) curve-fit.

**Blood pressure measurements**

Blood pressure was measured in the tail artery in pregnant C57BL/6 *Lgals3^+/+^* or *Lgals3^-/-^* females from E10 to E17 (n = 6-8). Measurements were performed with a computerized, non-invasive tail-cuff acquisition system (CODA System, Kent Scientific Corporation) as previously described ^2^. Briefly, for blood pressure measurements the mice were placed in plastic holders and the body temperature was maintained between 34 and 36 °C with infrared heating. The CODA system utilizes volume-pressure recording technology to detect changes in tail volume that correspond to systolic and diastolic pressures (mmHg) and calculates mean arterial pressure (MAP) during each cycle.

**Albumin-to-creatinine ratio (ACR)**

Twenty-four-hour urine samples were collected and creatinine and albumin levels on E17 were determined (Exocell, #1011 and #1012) following the manufacturer’s instructions. Briefly, urine samples were cleared by centrifugation before starting the assays. For creatinine determination the 5-fold diluted samples and the ready-to-use standards were added to a microtiter plate (Nunc-Immuno™ Plate, 456537) and the picrate working solution was incubated. After 10 minutes, the OD at 500 nm was determined (= OD_picrate_). The acid reagent was added and incubated for 5 minutes. The OD was measured at 500 nm (OD_picrate+acid_). The differences of OD_picrate_ and OD_picrate+acid_ were calculated for every sample and the creatinine levels were calculated from the standard curve. The albumin standards were prepared in a 2-fold dilution series (from 10 to 0.156 µg/ml). Standards and samples were added to the supplied test microtiter plates. The anti-albumin antibody was applied and incubated for 30 minutes at room temperature. After washing HRP-conjugated antibody was added and incubated for 30 min at RT. The plate was washed and incubated for 10 min with color developer. After addition of color stopper, the OD at 450 nm was determined and the albumin concentrations were calculated from the standard curve. For calculating the ACR expressing the amount of albumin (µg) per mg creatinine, the albumin concentrations were divided by the creatinine levels for every sample.

**Evaluation of kidney filtration capacity**

We injected intravenously 2.5 mg FITC-labeled dextran (Sigma-Aldrich, FD-2000S) 15 min before euthanizing the mice. Kidneys were frozen and cryostat sections (10 µm) were counter-stained with DAPI. The glomeruli in the kidneys were examined under the fluorescent microscope. Normally, the FITC-labeled dextran (MW 2,000,000) is filtered in the glomeruli from the blood, so that a green staining can be detected. Due to a reduced kidney filtration, which can be caused by endotheliosis, less FITC-labeled dextran is filtered.

**RNA isolation and quantitative real time RT-PCR (mouse)**

Total RNA was isolated from placental and whole implantation tissues with the RNeasy Protect Mini Kit (Qiagen, 74124). cDNA was generated with random primers (Invitrogen) after DNase digestion from 1 µg RNA in 25 µl and quantitative real time PCR was performed using the TaqMan 7500 System (Applied Biosystems). Each reaction had a total volume of 11 µl containing 1µl cDNA, 6.25 µl *Power* SYBR® Green PCR master mix (Applied Biosystems, 4367659), 3.75 µl DEPC water and 900 nM of the appropriate forward and reverse primers. The primer sequences are given in **Table 4**. The following PCR program was used: 2 min at 50°C, 10 min at 95°C, 40 cycles of 15 sec at 95°C and 60 sec at 60°C. Subsequently, a melting curve analysis was performed which consisted of 70 cycles of 10 seconds with a temperature increment of 0.5°C/cycle starting at 60°C. We calculated the relative expression (RE) with the following equation: RE = 2^-dCt^, in which Ct = Ct _gene of interest_ - Ct _reference gene_.

**RNA isolation and *Lgals3* quantitative real time RT-PCR (human)**

Total mRNA was isolated with a combined protocol of QIAzol lysis reagent and Qiagen RNeasy mini kit (including the RNase-Free DNAse set) (Qiagen) according to the manufacturer’s protocol for human placenta and decidua tissue. RNA quantity and quality were confirmed by RNA 6000 Chip in the 2100 Bioanalyzer (Agilent Technologies) and NanaDrop UV/VIS-Spectrometer (PeqLab). 2 µg RNA was reverse transcribed into cDNA by using the Transcriptor First Strand cDNA synthesis Kit from Roche Diagnostics and analyzed by real time qRT-PCR on ABI 7500 Fast sequence detection system (PE Biosystems). Gal-3 primer sequences were as follows: 5’ TCGCCAGCAACCTGAATCTC 3’ and 5’ GCACGAAGCTCTTAGCGTCA 3’; Endogenous-control Eukaryotic *18S* rRNA (GenBank accession number: X03205) (PE Biosystems) or *HPRT1* forward 5′-GTTTGTTGTAGGATATGCCCTTGAC-3′ and reverse 5′-GACTCCAGATGTTTCCAAACTCAAC-3′ were used for normalization.

**Angiotensin II receptor type 1 autoantibodies (AT1AA)**

AT1AA levels were determined in mouse serum samples as described ^6^. Briefly, the immunoglobulin fraction was isolated by ammonium sulfate precipitation. The precipitates were washed and dissolved in dialysis buffer. The procedure was repeated twice. The immunoglobulin fractions and the synthetic peptide corresponding to the sequence of the second extracellular loop of the AT1 receptor were mixed and added to cultured neonatal rat heart muscle cells at a concentration of 1:40. The mixtures were prepared as duplicates for each serum sample. The beating rate was counted for 15 seconds at six different points with in the culture flask to measure the chronotropic response of the heart muscle cells. To prove the AT1 antibody-specific positive response, 1 µM of the AT1 antagonist Losartan was added and the beating rate was re-analyzed for 15 seconds. If no chronotropic response was observed, 1 µM angiotensin II was added as a positive control and the beating rate was counted again in these samples.

**Gal-3 Western blotting**

Frozen placental tissue was lysed in lysis buffer containing 1% (v/v) Y30 and disaggregated using a gentleMACSDissociator (MiltenyiBiotec) as described previously ^1^. Ten µg of protein lysate was separated by SDS-PAGE under reducing conditions on 10% polyacrylamide gels and then transferred to PVDF membranes. Membranes were blocked for 1 hour at room temperature in 5% (v/v) skim milk and incubated for 15 hours at 4°C with primary antibody gal-3 (1:1000, Santa Cruz Biotechnology SC-20157) or β-actin (loading control) (1:15000, Sigma A5441). Membranes were incubated with goat anti-rabbit HRP (1:3000, Bio-Rad 1706515) or goat anti-mouse HRP (1:3000, Bio-Rad 1706516) secondary antibody, respectively, in 2% (v/v) skim milk for 1 hour at room temperature and signal was detected using the enhanced chemiluminescence detection system (PerkinElmer) and ImageQuant LAS 4000 (GE Healthcare). ImageJ software was used to quantify the densitometry of protein bands.

**Galectin-3 staining in human samples**

Immunohistochemistry was performed on formalin fixed, paraffin embedded placental tissues as previously described ^1^. Briefly, 4 µm sections derived from the FGR or normal pregnancy cohort were dewaxed and rehydrated through graded alcohols. Antigen retrieval was performed at 99°C for 20 minutes in a pH 9 retrieval solution and slides were incubated in Sequenza racks with primary antibody gal-3 (0.5 µg/ml; Santa Cruz Biotechnology sc-32790), cytokeratin 7 (0.09 µg/ml; Abcam ab68459), mouse IgG1 isotype control (Dako) or rabbit monoclonal antibody (Cell Signaling Technology) at equivalent concentrations at 4°C for 18 hours. Staining was visualized using the NovaRed peroxidase HRP substrate kit (Vector Laboratories) and counterstained using Mayer’s hematoxylin (Merck Millipore). The sections were imaged using a NanoZoomer-SQ Digital Slide Scanner (Hamamatsu) and NanoZoomer Digital Pathology software at 200x magnification and antibody staining was quantitated using ImageJ. A macro was specifically developed within Image J to detect red pigments, which coincided with the regions of the section expressing either gal-3 or CK7. A threshold was set in the software so that it would distinguish between the areas of red staining and all other areas to accurately quantitate protein expression. The same macro with the same optimized threshold was applied to all the images taken from all sections stained with antibody or isotype control. The software then determined the percentage area within the image that was stained red. The average percentage stained area of the three images taken per section, was calculated. Once the average percentage area for each section was calculated for gal-3 and CK7, a ratio of gal-3 to CK7 staining was calculated by dividing the total gal-3 staining by the amount of CK7 staining in sections taken from the sample tissue sample.

**References**

1 Powell, K. L. *et al.* Role for the thromboxane A2 receptor beta-isoform in the pathogenesis of intrauterine growth restriction. *Sci Rep* **6**, 28811, doi:srep28811 [pii]10.1038/srep28811 [doi] (2016).

2 Freitag, N. *et al.* Interfering with Gal-1-mediated angiogenesis contributes to the pathogenesis of preeclampsia. *Proceedings of the National Academy of Sciences of the United States of America* **110**, 11451-11456, doi:1303707110 [pii]10.1073/pnas.1303707110 (2013).

3 Plaks, V. *et al.* Functional phenotyping of the maternal albumin turnover in the mouse placenta by dynamic contrast-enhanced MRI. *Molecular imaging and biology* **13**, 481-492, doi:10.1007/s11307-010-0390-1 (2011).

4 Barnes, S. R. *et al.* ROCKETSHIP: a flexible and modular software tool for the planning, processing and analysis of dynamic MRI studies. *BMC medical imaging* **15**, 19, doi:10.1186/s12880-015-0062-3 (2015).

5 Yushkevich, P. A. *et al.* User-guided 3D active contour segmentation of anatomical structures: significantly improved efficiency and reliability. *NeuroImage* **31**, 1116-1128, doi:10.1016/j.neuroimage.2006.01.015 (2006).

6 Wallukat, G. *et al.* Patients with preeclampsia develop agonistic autoantibodies against the angiotensin AT1 receptor. *J Clin Invest* **103**, 945-952, doi:10.1172/JCI4106 [doi] (1999).

**Supplementary Figure Legends**

**Supplementary Figure 1** Additional representative images of galectin-3 (gal-3) and cytokeratin (CK) 7 immunohistochemistry in normal pregnancy (NP) and fetal growth restriction (FGR) placentae. Overview bar = 200 µm and high power bar = 100 µm.

**Supplementary Figure 2** Gal-3 levels remain unchanged in human PE patients and Lgals3^-/-^ mice do not display PE-like symptoms. **a***-***b** Real-time qPCR analysis of placental (**a**) and decidual (**b**) expression of gal-3 in control (n = 36), early (n = 18) and late (n = 17) onset PE patients. **c** Circulating gal-3 levels as analyzed by ELISA in early onset PE (<34 weeks), late onset PE (>34 weeks) and control (32-37 weeks) women were included in this analysis (n = 9-14). **d** Representative frozen-sections were prepared from control, early and late onset PE patients (n = 5) and stained for gal-3 by immunofluorescence (red: gal-1; blue: DAPI nuclear staining). Pictures were imaged at ×20 magnification on a confocal fluorescence microscope (bar = 100μm). **e** Systolic and diastolic blood pressure profiles as measured on E17 in Lgals3^+/+^ (WT) and Lgals3^-/-^ (KO) dams (left panel, n = 5). No differences were observed in the E17 heart-to-tibia ratio between Lgals3^+/+^ and Lgals3^-/-^ mice (right panel, n = 5-6). **f** To detect proteinuria, the albumin-to-creatinine ratio (ACR) was determined by ELISA in 24 h urine samples collected from E16 to E17 (left panel, n = 5). Representative images of maternal kidney perfusion as analyzed with FITC-dextran (green) on E17; white encircled glomeruli are shown (right panel, bar = 200 µm). **g** On E17, angiotensin II receptor type 1 autoantibodies (AT1AA) were measured in the serum of Lgals3^+/+^ WT and Lgals3^-/-^ KO dams (n = 3), by culturing the immunoglobulin fraction with neonatal rat heart muscle cells. The activation of the AT1 by autoantibodies increases the beating rate in these cells. **h** VEGF, sEng and sFlk-1 serum levels were analyzed by ELISA on E17 (n = 4). **i** Masson-Goldner’s trichrome stain was used to visualize spiral arteries in the decidua on E10 and E13. Quantification of the inner lumen-to-outer diameter ratio (LD/OD) of the spiral artery walls obtained from Lgals3^+/+^ WT and Lgals3^-/-^ KO dams (left bar = 500 μm and right bar = 50 µm, n = 10-32). In all figures, data are expressed as mean ± SEM. Data were analyzed using one-way ANOVA followed by Bonferroni´s test (**a**-**d**) and two-tailed *t* test (**e**-**i**).

**Supplementary Figure 3** High power magnification images. **a** Glycogen cells in the spongiotrophoblast of the placenta were stained with periodic acid Schiff (PAS) and counted in 3 squares (1x1 mm) per mouse on E13 (n = 14-21, bar = 50 µm). **b** Accumulation of mature NK cells (PAS^+^DBA^+^) in the decidua basalis were counted in 3 to 4 squares (1x1 mm) per mouse (n = 19-26, bar = 100 µm).

**Supplementary Tables**

**Table 1** Characteristics of the recruited participants at University Medical Center Hamburg-Eppendorf, Germany. Clinical characteristics are displayed for blood collected from gestational age matched control (normal term and pre-term) and FGR affected pregnancies. Data include maternal age (MA), body mass index (BMI), gestational age (GA) at delivery, birth weight and fetal gender. Data are represented as mean (SD). Independent samples T-test, ‡*P < 0.005*.

| Subgroup | MA (years) | BMI | GA (weeks) | Birth weight (g) | Fetal Gender (M/F) |
| --- | --- | --- | --- | --- | --- |
| Healthy  Controls  (*n*=33) | 32.0 (3.7) | 23.0 (3.1) | 39.9 (1.05) | 3603.0 (317.8) | 57.6%/42.4% |
| FGR  (*n*=32) | 31.7 (2.8) | 22.9 (3.1) | 39.5 (0.99) | 2802.5 (221.6)‡ | 46.9%/53.1% |

**Table 2** Characteristics of the recruited participants at the Kolling Institute, Sydney, Australia. Clinical characteristics are displayed for placental tissue collected from gestational age matched control (normal term and pre-term) and FGR affected pregnancies. Data include maternal age (MA), body mass index (BMI), systolic (SBP) and diastolic (DBP) blood pressure, gestational age (GA) at delivery, birth weight and fetal gender. Data are represented as mean (SD). Independent samples T-test, ‡*P < 0.005*.

| Subgroup | MA (years) | BMI | SBP  (mm/Hg) | DBP  (mm/Hg) | GA (weeks) | Birth weight (g) | Fetal Gender  (M/F) |
| --- | --- | --- | --- | --- | --- | --- | --- |
| Healthy  Controls  (*n*=27) | 33.0 (4.1) | 23.6 (4.6) | 111.1 (15.7) | 69.0 (9.0) | 37.9 (3.2) | 3222.4 (750.9)‡ | 74.1%/25.9% |
| FGR  (*n*=19) | 34.7 (3.6) | 23.0 (3.1) | 117.1 (12.3) | 74.5 (8.6) | 36.5 (2.8) | 1930.5 (717.9) | 36.8%/63.2% |

**Table 3** Characteristics of the recruited participants at Oslo University Hospital, Norway. Clinical characteristics are displayed for placental tissue and maternal blood collected from gestational age matched control and early or late preeclampsia (Early PE or Late PE) affected pregnancies. Data include maternal age (MA), systolic (SBP) and diastolic (DBP) blood pressure, gestational age (GA) at delivery, placenta weight (g) and sFlt-1: PlGF ratio. sFlt-1: soluble fms-like tyrosine kinase 1; PlGF: placental growth factor. Maternal serum levels of sFlt-1 and PlGF were both measured in pg/mL. Data are represented as mean (SD). Independent samples T-test, **P<0.05,* ‡*P < 0.005*.

| Subgroup | MA (years) | SBP  (mm/Hg) | DBP  (mm/Hg) | GA (weeks) | Placenta weight (g) | sFlt-1: PlGF ratio |
| --- | --- | --- | --- | --- | --- | --- |
| Healthy  Controls  (*n*=36) | 32.1 (4.8) | 122.9 (16.1) | 71.3 (7.5) | 38.4 (0.5) | 586.5 (128.9) | 68.9 (4.8) |
| Early PE  (*n*=18) | 32.7 (3.2) | 174.1 (24.0) | 100.2 (10.4) | 30.2 (2.9) | 243.8 (151.5) | 808.7 (174.0)‡ |
| Late PE  (*n*=18) | 31.5 (5.5) | 160.1 (16.2) | 98.2 (6.2) | 37.9 (0.8) | 595.4  (201.8) | 158.2 (47.6)* |

**Table 4** Sequences of mouse quantitative real time RT-PCR primers used in the present study.

| **Gene** | **Forward primer (5’-3’)** | **Reverse primer (5’-3’)** |
| --- | --- | --- |
| *TGF-β* | TTGCTTCAGCTCCACAGAGA | TGGTTGTAGAGGGCAAGGAC |
| *IL-10* | CAGAGCCACATGCTCCTAGA | TGTCCAGCTGGTCCTTTGTT |
| *IFN-Ƴ* | TAGCCAAGACTGTGATTGCGG | AGACATCTCCTCCCATCAGCAG |
| *TNF-α* | AAGCCTGTAGCCCACGTCGTA | AGGTACAACCCATCGGCTGG |
| *IL-17* | TCCAGAAGGCCCTCAGACTA | AGCATCTTCTGACCCTGAA |
| *Hand 1* | ATCATCACCACTCACACCCG | CTCTGGAAGTAAGGCCGCTC |
| *Prl2c2* | AGCCAGGCTCACACACTATT | ACTAGATCGTCCAGAGGGCT |
| *Prl3d1* | GGCCGCAGATGTGTATAGGG | AGTTTCGTGGACTTCCTCTCG |
| *Ascl2* | GTGAAGGTGCAAACGTCCAC | CCCTGCTACGAGTTCTGGTG |
| *Junb* | AGGCAGCTACTTTTCGGGTC | TTGCTGTTGGGGACGATCAA |
| *Gab1* | ATTTCCACCGTGGATTTGAAC | GATCTATCGCTCGGAAAGGTC |
| *Gcm1* | AAGCTTATTCCCTGCCGAGG | AAAGATGAAGCGTCCGTCGT |
| *Gapdh* | TGACGTGCCGCCTGGAGAAA | AGTGTAGCCCAAGATGCCCTTCAG |
