# Supplementary figures and images for: Galectin-3 deficiency in pregnancy increases the risk of fetal growth restriction (FGR) via placental insufficiency
Source: Cell Death Dis. 2020 Jul 23;11(7):560. doi: 10.1038/s41419-020-02791-5 (PMC7378206; doi:10.1038/s41419-020-02791-5)

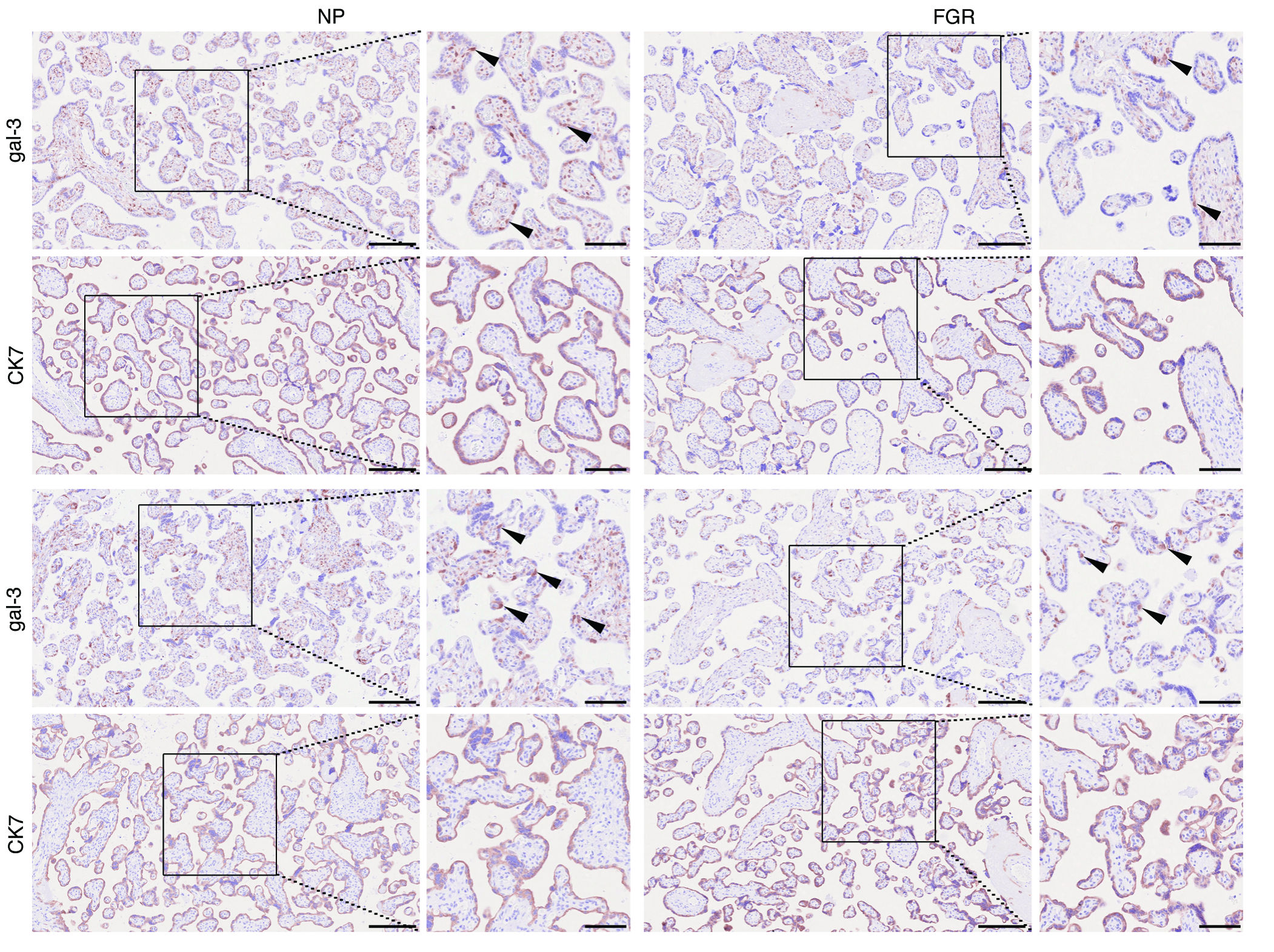

Supplement: Supplementary file 2 — Supplementary information 2 [file 41419_2020_2791_MOESM2_ESM.tif]

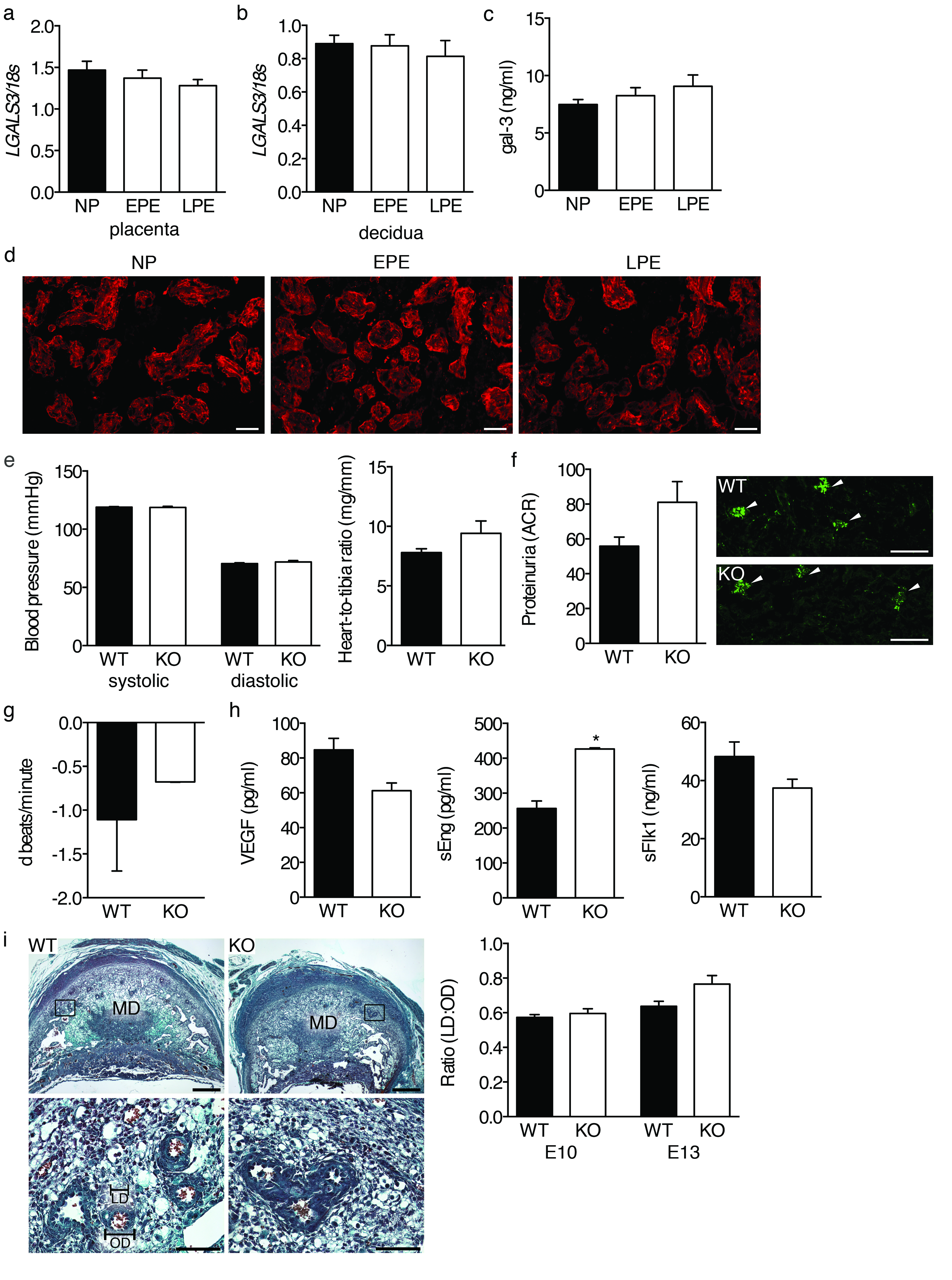

Supplement: Supplementary file 3 — Supplementary information 3 [file 41419_2020_2791_MOESM3_ESM.tif]

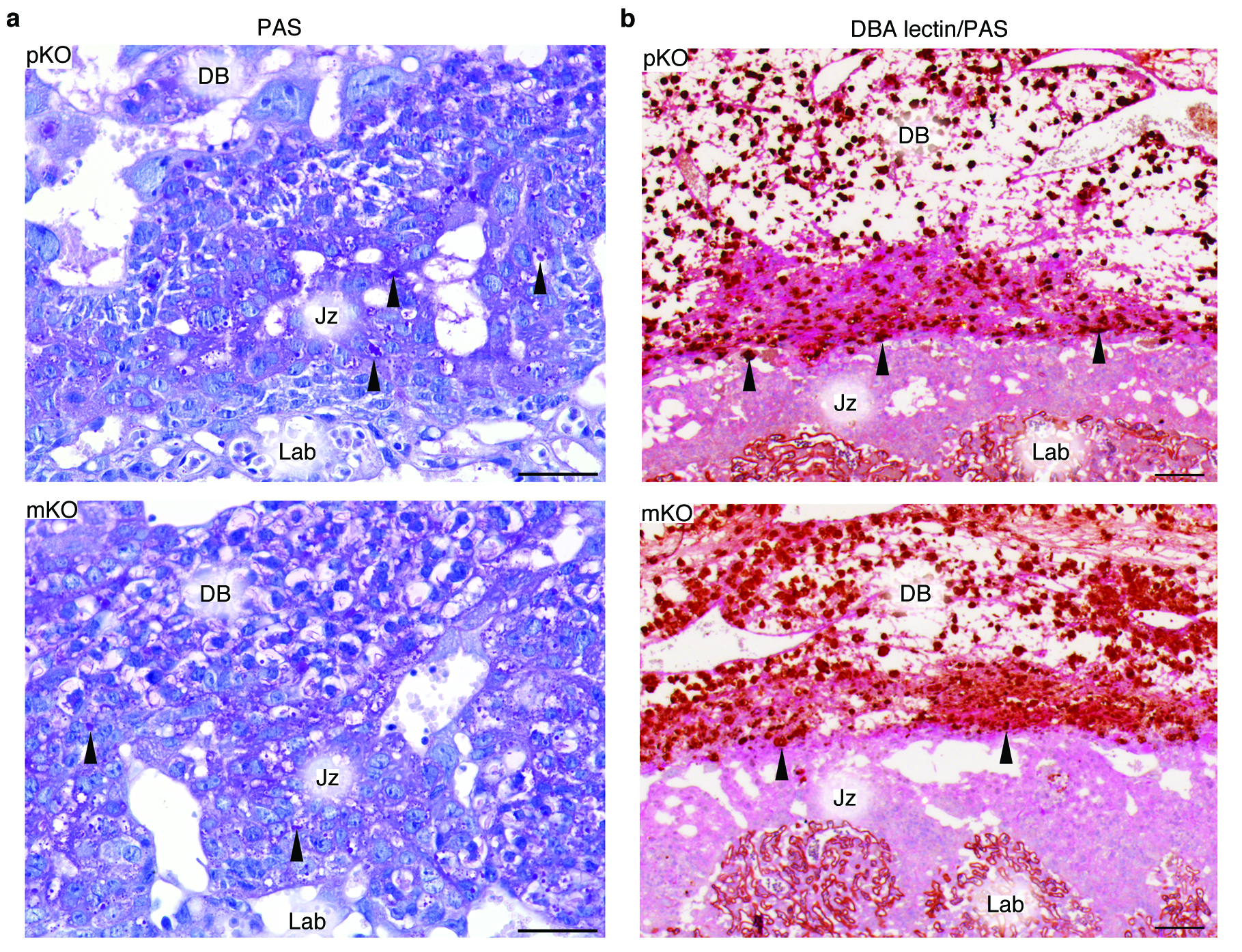

Supplement: Supplementary file 4 — Supplementary information 4 [file 41419_2020_2791_MOESM4_ESM.tif]
